# The CXCL9/10/11-CXCR3 axis as a predictor of COVID-19 progression: a prospective, case-control study

**DOI:** 10.1590/0037-8682-0128-2023

**Published:** 2023-07-24

**Authors:** Neslihan Çelik, Onur Çelik, Esra Laloğlu, Alev Özkaya

**Affiliations:** 1 Health Sciences University, Erzurum Regional Education and Research Hospital, Department of Infection Diseases and Clinical Microbiology, Erzurum, Turkey. Health Sciences University Erzurum Regional Education and Research Hospital Department of Infection Diseases and Clinical Microbiology Erzurum Turkey; 2 Health Sciences University, Erzurum Regional Education and Research Hospital, Department of Chest Diseases, Erzurum, Turkey. Health Sciences University Erzurum Regional Education and Research Hospital Department of Chest Diseases Erzurum Turkey; 3 Ataturk University School of Medicine, Department of Biochemistry, Erzurum, Turkey. Ataturk University School of Medicine Department of Biochemistry Erzurum Turkey; 4 Health Sciences University, Erzurum Regional Education and Research Hospital, Department of Biochemistry, Erzurum, Turkey. Health Sciences University Erzurum Regional Education and Research Hospital Department of Biochemistry Erzurum Turkey

**Keywords:** COVID-19, CXCL9/10/11-CXCR3 axis, Prognosis

## Abstract

**Background:**

This study examined the relationship between levels of the chemokines CXCL9, CXCL10, CXCL11, and CXCR3 and mortality in patients with COVID-19..

**Methods:**

A total of 71 patients hospitalized with COVID-19 and 35 health workers with no symptoms and negative SARS-CoV-2 PCR results were included in the study. CXCL9, CXCL10, CXCL11, and CXCR3 levels were measured in blood samples using enzyme-linked immunosorbent assays. Participants were divided into three groups: healthy individuals, patients with mild to moderate pneumonia, and patients with severe pneumonia. Patients were also divided into sub-groups according to the outcome: dead and survived.

**Results:**

Serum CXCL9, CXCL10, CXCL11, and CXCR3 levels were significantly higher in patients with severe COVID-19 than in those with non-severe COVID-19; were higher in both patient groups than in the control group; and were higher in patients who died than in those who survived. Lymphocyte counts, and fibrinogen and PaO_2_/FiO_2_ levels were significantly lower in patients with severe COVID-19 than in those with moderate disease. Patients with COVID-19 also had elevated neutrophil/lymphocyte ratios, neutrophil counts, and lactate dehydrogenase, C-reactive protein, D-dimer, and ferritin levels.

**Conclusions:**

This study confirmed that CXCL9, CXCL10, CXCL11, and CXCR3 levels are associated with disease severity in patients with COVID-19. These laboratory parameters can help to estimate disease severity and predict outcomes, and are useful in clinical decision-making.

## INTRODUCTION

COVID-19 is a potentially fatal viral disease, ranging in severity from mild upper airway infection to severe pneumonia, acute respiratory distress syndrome (ARDS), and multiple organ failure^1^.

A hyperinflammatory host response plays an important role in the manifestation of severe disease^2^. Overexpression of proinflammatory chemokines and cytokines involved in the cytokine storm is the source of the pulmonary complications caused by the virus^3^.

Cytokines are a category of proteins, peptides, and glycoproteins secreted by hematopoietic and non-hematopoietic cells that respond to different types of stimuli. They are categorized into five groups: the interleukin-1, hematopoietin, interferon, tumor necrosis factor, and chemokine families^1^.

Chemokines are important proteins involved in the regulation of leukocyte migration in peripheral lymphatic organs and play an important role in the inflammatory response. They cannot be detected in most nonlymphoid tissues under physiological conditions. Their production by blood and tissue cells is strongly induced by interferon gamma (IFN-γ), the most typical T helper 1 (Th1) cytokine in several diseases^4^.

Generally, chemokines contribute to combating viral infections by recruiting immune cells to the locus of infection, increasing the cytotoxic activities of these cells, and encouraging the release of antiviral mediators^5^.

CXCL9, CXCL10, and CXCL11 are three chemokines without the Glu-Leu-Arg (ELR) motif. Due to their approximately 40% amino acid sequence similarity, they are more closely related than other chemokines. These chemokines play a specific role in the Th1 response^4^. Because they have a common receptor (CXCR3), CXCL9, CXCL10, and CXCL11 are often studied together, and studies have been conducted on their combined effect in infection, injury, and immunoinflammatory responses^6^.

The primary aim of this study was to investigate the association between mortality and the CXCL9, CXCL10, CXCL11, and CXCR3, chemokine levels, which contribute to the pathogenesis of COVID-19 through hyperinflammation, in patients with COVID-19.

## METHODS

Seventy-one patients with COVID-19 hospitalized in the COVID-19 ward of our hospital between January and September 2021 and 35 healthcare workers with negative reverse-transcription polymerase chain reaction (RT-PCR) SARS-CoV-2 test results were included in this study. Patients aged older than 18 years were enrolled; however, those with other acute or chronic systemic disease, hematologic disease, malignancy, or immunosuppressive drug use were excluded. The diagnosis of COVID-19 was confirmed by a positive SARS-CoV-2 RT-PCR results on a nasal swab specimen, or chest radiography and computed tomography findings compatible with COVID-19. This study was conducted in accordance with the principles of the Declaration of Helsinki and was approved by the Health Sciences University Erzurum Regional Education and Research Hospital Clinical Research Ethical Committee, Türkiye (2022/08-108).

The 106 individuals enrolled in this study were assigned to three groups. The first group consisted of healthy PCR-negative healthcare workers who consented to participate (n = 35). The second group consisted of patients with moderately severe pneumonia (n = 30). The third group consisted of patients with severe pneumonia (respiratory rate ≥ 30 breaths/min, SpO_2_ ≤ 92%, and/or pulmonary infiltration > 50%) (n = 41). The patients were also subdivided into those who died and those who survived. Two groups were also established based on the extent of lung involvement on imaging: mild/moderate involvement and severe involvement. The extent of pulmonary involvement was classified as minimal (1-5%), mild (6-25%), moderate (26-49%), severe (50-74%), and diffuse (≥ 75%). Serum levels of CXCL9, CXCL10, CXCL11, and CXCR3 were compared between groups.

Patients were followed up in accordance with the Turkish Ministry of Health guidelines for care and treatment of COVID-19 in adults. Patients with refractory fever despite treatment, continuously rising C-reactive protein (CRP) and ferritin levels, D-dimer elevation, lymphopenia or thrombocytopenia, abnormal liver function tests, or hypofibrinogenemia were monitored for macrophage activation syndrome (MAS).

Blood specimens were centrifuged for 15 min at 4000 rpm to separate the serum. The serum samples were stored in Eppendorf tubes at −80ºC. CXCL9, CXCL10, CXCL11, and CXCR3 levels were measured in the same blood specimen and recorded. Laboratory test results, including serum CRP, lactate dehydrogenase (LDH), aspartate aminotransferase (AST), alanine aminotransferase (ALT), alkaline phosphatase (ALP), gamma-glutamyltransferase (GGT), creatinine, D-dimer, troponin I, prothrombin time (PT), fibrinogen, ferritin, white blood cell (WBC), lymphocyte count, neutrophil count, neutrophil/lymphocyte ratio, and platelet count were also recorded.

Serum CXCL9, CXCL10, CXCL11, and CXCR3 levels were measured using an enzyme-linked immunosorbent assay (Bioassay Technology Laboratory, Shanghai, China). Serum LDH, AST, ALT, ALP, GGT, CK, creatinine, and CRP levels were measured using commercial kits and a Beckman Coulter AU5821 analyzer (Brea, CA, USA). Ferritin and troponin I levels were measured using commercial kits and a Beckman Coulter DxI800 analyzer. WBC, lymphocyte, neutrophil, and platelet counts were measured using a Sysmex XN-9000 (Kobe, Japan) analyzer. D-dimer levels were measured using a Radiometer AQT90 flex analyzer (Copenhagen, Denmark). The activated partial thromboplastic time, international normalized ratio, and fibrinogen level were measured using a Stago STA R Max analyzer (Stago, Asnières sur Seine, France).

### Statistical analysis

Data were analyzed using SPSS for Windows (version 20.0; IBM Corp., Armonk, NY, USA). Categorical variables were expressed as frequencies and percentages, and continuous variables were expressed as means ± standard deviations. Visual (histogram) and analytical methods (Kolmogorov-Smirnov or Shapiro-Wilk tests, as appropriate) were used to assess the normality of the distribution of continuous variables. Chi-square tests were used to compare categorical variables between groups, and t-tests or one-way analysis of variance were used to compare continuous variables between groups, as required. The significance of intergroup differences was evaluated using post-hoc Tukey tests. Receiver operating characteristic (ROC) curves were plotted, and the optimal cut-off values sensitivity, specificity, and area under the curve (AUC) were calculated to assess the predictive power of different measures. Statistical significance was set at 5%.

## RESULTS

Seventy-one patients with COVID-19 and 35 healthy controls were included in the study. The mean age was 65.8 ± 14.5 years in the patient group and 62.6 ± 12.3 years in the control group. No significant differences in age (p = 0.41) or sex (p = 0.23) were observed between the groups.

The COVID-19 group consisted of 45 men (63%) and 26 women (37%). Among the men, 21 (47%) had moderate COVID-19 and 24 (53%) had severe COVID-19. Among the women, 9 (35%) had moderate COVID-19 and 17 (65%) had severe COVID-19. COVID-19 severity did not differ significantly by sex (p = 0.07). The mean age was 65.53 ± 15.89 years in patients with moderate disease and 66.05 ± 13.12 years in those with severe disease. COVID-19 severity did not differ significantly by age (p = 0.88).

In the COVID-19 group, the survivors were aged 65.74 ± 12.92 years, and those who died were aged 65.92 ± 16.22 years. Men comprised 27 (64%) of the survivors and 19 (66%) of the patients who died. No differences were observed in COVID-19 severity by age or sex (p = 0.948 and p = 0.915, respectively).

The laboratory data of patients with COVID-19 are compared by disease severity in Table 1.The lymphocyte count, and fibrinogen and PaO_2_/FiO_2_ levels were all significantly lower in patients with severe COVID-19 than in those with moderate disease (all p < 0.001). The neutrophil/lymphocyte ratio (NLR), neutrophil count, and LDH, CRP, D-dimer, and ferritin levels were all significantly higher in patients with severe COVID-19 than in those with moderate disease (p < 0.001, p < 0.03, p < 0.001, p < 0.001, p < 0.006, and p < 0.001, respectively).

**TABLE 1:** Comparison of age and laboratory data on admission in patients with moderate and severe COVID-19.

Parameter	Reference value	Moderate Mean [SD]	Severe Mean [SD]	p
		n=30	n=41	
Age (years)		65.5 [15.9]	66.1 [13.1]	0.882
WBC (cells/µL)	4390-11590	9446 [4120]	14750 [5438]	**0.001**
Lymphocytes (cells/µL)	1210-3770	958 [454]	560 [316]	**0.001**
Neutrophils (cells/µL)	2100-8800	7542 [4187]	9345 [4016]	**0.03**
NLR		9.4 [7.1]	23.2 [15.6]	**0.001**
AST (U/L)	13-40	73.2 [42.3]	66.0 [36.5]	0.857
ALT (U/L)	7-40	106.7 [65.4]	96.9 [73.2]	0.854
LDH (U/L)	120-246	422.3 [125.2]	620.7 [176.1]	**0.001**
GGT (U/L)	0-73	76.1 [64.9]	72.3 [68.2]	0.124
ALP (U/L)	46-116	144.1 [53.8]	136.7 [48.5]	0.825
Creatinine (mg/dL)	0.7-1.3	1.2 [0.7]	1.0 [0.6]	0.75
Prothrombin time (s)	12-16	13.2 [1.7]	14.5 [4.2]	**0.06**
CRP (mg/dL)	0-5	56.7 [45.2]	146.5 [82.4]	**0.001**
Troponın (ng/dL),	0-45	24.7 [62.5]	36.4 [45.3]	0.125
PaO_2_/FiO_2_		265.7 [84.3]	164.2 [28.5]	**0.001**
D-dimer (ng/mL)	0-500	1250,4 [1672.3]	2944.8 [4550.1]	**0.00**6
Ferritin (ng/mL)	22-322	614.8 [362.4]	1450.2 [316.5]	**0.001**
Fibrinogen (ng/mL)	200-400	530.5 [154.6]	412.5 [130.8]	**0.001**

**ALP:** alkaline phosphatase; **ALT:** alanine aminotransferase; **AST:** aspartate aminotransferase; **CRP:** C-reactive protein, **GGT:** gamma glutamyl transferase; **LDH:** lactate dehydrogenase; **NLR:** neutrophil/lymphocyte ratio; **PaO**_2_ **/FiO**_2_ **:** ratio of the partial pressure of arterial oxygen to the fraction of inspired oxygen; **WBC:** white blood cell.

The CXCL9, CXCL10, CXCL11, and CXCR3 levels in the COVID-19 group and healthy controls are compared in Table 2**.** CXCL9, CXCL10, CXCL11, and CXCR3 levels were significantly higher in patients with severe COVID-19 group than in those with moderate disease. CXCL9, CXCL10, CXCL11, and CXCR3 levels were significantly higher in both patient groups than in the control group.

**TABLE 2:** Comparison of CXCL9, CXCL10, CXCL11, CXCR3 levels between patients with moderate and severe COVID-19 and healthy controls.

Parameter	Moderate disease Mean [SD]	Severe disease Mean [SD]	Healthy controls average [SD]	p
	n=30	n=41	n=35	
CXCL9 (pg/mL)	704.55 [471.14]	940.09 [498.83]	194.64 [88.13]	**<0.043** ^a^ **<0.001** ^b,c^
CXCL10 (pg/mL)	159.62 [43.63]	288.66 [102.71]	71.01 [39.77]	**<0.001** ^a,b,c^
CXCL11 (pg/mL)	139.64 [67.58]	290.52 [60.23]	93.49 [54.29]	**<0.001** ^a,b^ **<0.008** ^c^
CXCR3 (pg/mL)	559.38 [234.64]	896.85 [326.77]	163.48 [52.30]	**<0.001** ^a,b,c^

**Note:** Statistically significant results are indicated in bold. ^a^ Patients with moderate vs. severe COVID-19; ^b^ Patients with severe COVID-19 vs. controls; ^c^ Patients with moderate COVID-19 vs. controls.

The CXCL9, CXCL10, CXCL11, and CXCR3 levels were significantly associated with clinical manifestations including fever, tachypnea, muscle/joint pain, sore throat, headache, somnolence, shortness of breath, and nausea/vomiting (Table 3).

**TABLE 3:** Comparison of serum CXCL9, CXCL10, CXCL11 and CXCR3 levels in patients with COVID-19 according to the clinical manifestations.

Symptom	CXCL9 Mean [SD]	CXCL10 Mean[SD]	CXCL11 Mean [SD]	CXCR3 Mean [SD]	P*
	(pg/mL)	(pg/mL)	(pg/mL)	(pg/mL)	
Fever					p^a^=0.016 p^b,c,d^<0.001
Yes	957 [494]	278 [110]	278 [76]	876 [340]	
No	672 [461]	171 [51]	153 [78]	578 [237]	
Tachypnea					p^a^=0.025 p^b,c,d^<0.001
Yes	950 [510]	280 [109]	281 [74]	887 [339]	
No	683 [442]	168 [46]	149 [72]	561 [216]	
Muscle or joint pain					p^a^=0.028 p^b,c,d^<0.001
Yes	948 [501]	281 [108]	280 [75]	878 [346]	
No	685 [459]	166 [45]	150 [74]	575 [221]	
Sore throat					p^a^=0.045 p^b,c,d^<0.001
Yes	703 [469]	169 [50]	155 [76]	590 [232]	
No	941 [500]	282 [109]	279 [76]	875 [349]	
Headache					p^a^=0.012 p^b,c,d^<0.001
Yes	970 [543]	287 [106]	286 [69]	902 [341]	
No	673 [380]	166 [47]	151 [75]	564 [209]	
Somnolence					p^a^=0.026 p^b,c,d^<0.001
Yes	949 [503]	281 [108]	283 [71]	283 [71]	
No	683 [454]	167 [47]	146 [72]	146 [72]	
Shortness of breath					p^a^=0.036 p^b,c,^<0.001 p^d^=0.001
Yes	940 [509]	275 [109]	275 [73]	857 [300]	
No	687 [447]	171 [57]	153 [85]	597 [329]	
Nausea/vomiting					p^a^=0.005 p^b,c,d^<0.001
Yes	648 [419]	166 [49]	149 [70]	559 [218]	
No	981 [508]	284 [107]	283 [74]	897 [335]	

* p <0.05 was regarded as statistically significant. ^a^ for CXCL9 level, ^b^ for CXCL10 level, ^c^ for CXCL11 level, ^d^ for CXCR3 level.

The patients with COVID-19 were also divided into two groups according to the severity of pulmonary involvement on chest imaging. The first group consisted of patients with mild involvement, and the second group consisted of patients with severe involvement. CXCL9, CXCL10, CXCL11, and CXCR3 levels were compared between the two groups (Table 4). CXCL9, CXCL10, CXCL11, and CXCR3 levels were significantly higher in patients with severe pulmonary involvement than in those with mild to moderate involvement.

**TABLE 4:** CXCL9, CXCL10, CXCL11, and CXCR3 levels according to the severity of lung involvement on imaging.

Parameter	Mild to moderate involvement	Severe involvement	p
	Mean [SD]	Mean [SD]	
CXCL9 (pg/mL)	596 [223]	1351 [529]	<0.001
CXCL10 (pg/mL)	196 [53]	314 [138]	<0.001
CXCL11 (pg/mL)	190 [79]	304 [90]	<0.001
CXCR3 (pg/mL)	604 [184]	1068 [363]	<0.001

The patients were further classified into two groups based on the outcome: died or survived. Patients who died were more likely to develop MAS during follow-up. CXCL9, CXCL10, CXCL11, and CXCR3 levels were significantly higher in patients who died (Table 5).

**TABLE 5:** The relationship between CXCL9,CXCL10, CXCL11, CXCR3 levels and mortality.

Parameter	Survivors Mean [SD]	Non-survivors Mean [SD]	p
	n=42	n=29	
CXCL9 (pg/mL)	613 [200]	1170 [610]	< 0.001
CXCL10 (pg/mL)	206 [50]	275 [144]	< 0.005
CXCL11 (pg/mL)	210 [77]	251 [120]	< 0.08
CXCR3 (pg/mL)	630 [171]	934 [425]	< 0.001

**FIGURE 1:**
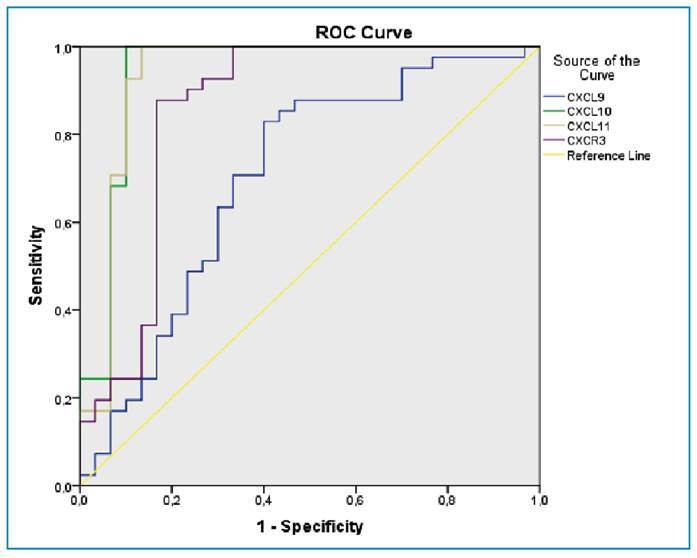
Receiver operating characteristic (ROC) curve analysis of CXCL9, CXCL10, CXCL11, CXCR3 in distinguishing between patients with moderate and severe COVID-19.

In the ROC curve analysis of the ability of CXCL9, CXCL10, CXCL11, and CXCR3 to discriminate between moderate and severe COVID-19, the AUC values were 0.702, 0.939, 0.933, and 0.857, respectively. Using cut-off values of 689.1, 228.4, 248.1, and 688.1 pg/mL according to the Youden index elicited sensitivity values of 63%, 83%, 81%, and 78% and specificity values of 70%, 90%, 90%, and 83%, respectively (Figure 1).

## DISCUSSION

In addition to their function in the regulation of leukocyte migration, chemokines and their receptors are involved in a broad range of pathophysiological processes such as inflammation, infectious diseases, allergic reactions, autoimmune diseases, and cancer^7^. CXCL9, CXCL10, and CXCL11 are members of the ELR-CXC chemokine family. Because these three chemokines possess a common receptor (CXCR3), they are generally studied together. Recent studies have revealed the pathogenic roles of CXCR3 and its ligands in several human inflammatory diseases^6^^,^^8^.

COVID-19 is a highly heterogeneous viral disease, and is characterized by a clinical course ranging from asymptomatic to fatal. Once the pathological role of the cytokine storm during COVID-19 became recognized, studies investigated whether specific chemokines may play a role in its progression^9^. This study is one such example. The levels of CXCL9, CXCL10, CXCL11, and CXCR3 were significantly higher in patients with COVID-19 than in healthy controls. The levels of these chemokines and their receptors were higher in patients with severe pneumonia than in those with moderate pneumonia. CXCL9, CXCL10, CXCL11, and CXCR3 levels were also significantly higher in patients who died than in those who survived.

CXCL10 can be released by different cells, including monocytes, leukocytes, and endothelial and epithelial cells. CXCL10 is elevated in several diseases, including hepatitis B, tuberculosis, cancer, diabetes, and autoimmune disorders^6^. CXCL10 is the most widely studied chemokine in patients with COVID-19. It has consistently been found to play a principal role in the cytokine storm induced by COVID-19^10^. High CXCL10 levels are strongly associated with the infiltration of immune cells into the alveolar space, peribronchial, and perivascular regions, and pulmonary damage^3^^,^^11^. Significantly higher serum concentrations of CXCL10 and viral loads in patients with COVID-19 have been reported in patients with fatal disease than in survivors, and a CXCL10 levels are positively correlated with the SARS-CoV-2 viral load^12^. The levels of various chemokines in the circulation have been examined in patients with severe and critical COVID-19, and CXCL10 levels have been described as biomarkers that are positively correlated with both disease severity and the risk of death^13^.

In agreement with previous studies, this study found higher CXCL10 levels in patients with severe pneumonia than in patients with moderate pneumonia, and significant elevation in patients developing MAS and fatal cases. CXCL10 levels have also been shown to be increased in patients with ARDS. However, higher CXCL10 levels were found in patients with ARDS manifestations thought to be of viral origin than in those with ARDS of suspected bacterial origin^12^. Studies of patients with hepatitis C have shown elevated CXCL9 and CXCL10 levels in patients with high viral loads, and that these decrease after treatment^14^^,^^15^. In patients with HIV infection, CXCL9 and CXCL10 levels are positively correlated with the viral load, and negatively correlated with the CD4^+^ T-cell count^6^. These studies suggest that CXCL10 and other chemokines are potential biomarkers of other viral infections.

CXCL9 is induced by IFN-γ in macrophages and is involved in cancer and viral infections. It is also thought to play a role in T-cell trafficking, chemotaxis, and activation^6^. Patients with severe COVID-19 have been reported to exhibit higher serum CXCL9 levels than those with mild disease^16^^,^^17^.

CXCL11 is expressed at high levels in peripheral blood leukocytes, the pancreas, and the liver. Of CXCL9, CXCL10, and CXCL11, CXCL11 exhibits the greatest affinity for CXCR3, followed by CXCL10 and CXCL9^18^. Elevated CXCL11 levels have also been observed in mixed cryoglobulinemia, Graves’ disease, and autoimmune diseases. CXCL11 levels are correlated with the CD4^+^ T-cell count in these diseases^6^^,^^19^^,^^20^^,^^21^.

CXCR3 is a receptor that is preferentially expressed on the surfaces of monocytes, T cells, NK cells, dendritic cells, and cancer cells^19^. All three chemokines are ligands for CXCR3, recruit CD4^+^ Th1 cells and CD8^+^ T cells to the site of tissue injury, and inhibit angiogenesis during pulmonary fibrosis. All three chemokines have been implicated in the pathogenesis of idiopathic pulmonary fibrosis. Most patients with severe COVID-19 develop pneumonia and hyperinflammation, probably as a result of the MAS, known as a cytokine storm. Pulmonary fibrosis occurs as a secondary event linked to the progression of pathology associated with the inflammatory response^20^.

In this study, the levels of these chemokines were compared according to the severity of pulmonary involvement. Significant elevations in the levels of all three chemokines and their receptors were observed in the severe pulmonary involvement group. This finding suggests that these factors may be responsible for the development of pulmonary fibrosis in patients with severe COVID-19.

Increases occur in parameters such as AST, ALT, urea, creatinine, LDH, creatine kinase, troponin, and CK-MB occur secondary to organ damage as the disease progresses and organ dysfunction develops. As in several other infections, an increase occurs in the CRP and ferritin levels, and the erythrocyte sedimentation rate as the disease progresses, and this has been linked to severe lymphopenia, thrombocytopenia, and leukopenia^21^^,^^22^^,^^23^.

The results of this study are consistent with the results of previous studies in terms of most parameters. Lymphocyte counts, fibrinogen levels, and PaO_2_/FiO_2_ levels were significantly lower in patients with severe COVID-19, whereas WBC, NLR, LDH, CRP, D-dimer, PT, and ferritin values were significantly higher in those with severe disease.

In this study, CXCL9, CXCL10, CXCL11, and CXCR3 levels showed higher specificity than sensitivity based on the Youden index. This suggests that these chemokines may be effective in predicting the course of severe COVID-19.

Interferon-inducible chemokines such as CXCL9, CXCL10, and CXCL11 may be vital to the host’s response in viral respiratory infections by encouraging viral elimination before the activation of the adaptive immune system^24^. Cytotoxic reactions associated with inflammatory chemokines sometimes exacerbate disease severity and result in tissue injury. These chemokines may have beneficial or detrimental effects in viral infections involving pulmonary tissues, and their inhibition is recommended as a viable therapeutic strategy for treating some viral respiratory infections^5^.

The principal limitation of this study is the absence of a control group of patients with other illnesses associated with pneumonia, such as influenza or bacterial pneumonia. Such a control group would clarify whether the observed differences in the humoral immune response are specific to SARS-CoV-2. Further studies involving control groups are required to determine the usefulness of these chemokines in identifying COVID-19.

In conclusion, identifying potential targets for reducing chemokine induction could be an important strategy for treating viral respiratory infections. This study emphasizes the association between CXCL9, CXCL10, CXCL11, and CXCR3 levels and disease severity in patients with COVID-19. It shows that these can predict disease severity and outcome and could assist in clinical decision-making. These findings may serve as a guide for future research. This study contributes to the literature by confirming the relationship between chemokine levels and fatal COVID-19 in a more accurate manner, and by supporting previous studies on the subject.
